# Robust 3D Skeletal Joint Fall Detection in Occluded and Rotated Views Using Data Augmentation and Inference–Time Aggregation

**DOI:** 10.3390/s25216783

**Published:** 2025-11-06

**Authors:** Maryem Zobi, Lorenzo Bolzani, Youness Tabii, Rachid Oulad Haj Thami

**Affiliations:** 1 ADMIR Laboratory, National School of Computer Science and Systems Analysis, Mohammed V University, Rabat 10000, Morocco; youness.tabii@ensias.um5.ac.ma (Y.T.); rachid.ouladhajthami@ensias.um5.ac.ma (R.O.H.T.); 2 flAIght, Viale dell’Innovazione 13, 20126 Milan, Italy; lorenzo.bolzani@flaight.it

**Keywords:** fall detection, 3D skeletal joints, data augmentation, graph convolutional networks, occlusion, rotated views, MMPose, Kinect, VIRA-GCN

## Abstract

Fall detection systems are a critical application of human pose estimation, frequently struggle with achieving real-world robustness due to their reliance on domain-specific datasets and a limited capacity for generalization to novel conditions. Models trained on controlled, canonical camera views often fail when subjects are viewed from new perspectives or are partially occluded, resulting in missed detections or false positives. This study tackles these limitations by proposing the Viewpoint Invariant Robust Aggregation Graph Convolutional Network (VIRA-GCN), an adaptation of the Richly Activated GCN for fall detection. The VIRA-GCN introduces a novel dual-strategy solution: a synthetic viewpoint generation process to augment training data and an efficient inference-time aggregation method to form consensus-based predictions. We demonstrate that augmenting the Le2i dataset with simulated rotations and occlusions allows a standard pose estimation model to achieve a significant increase in its fall detection capabilities. The VIRA-GCN achieved 99.81% accuracy on the Le2i dataset, confirming its enhanced robustness. Furthermore, the model is suitable for low-resource deployment, utilizing only 4.06 M parameters and achieving a real-time inference latency of 7.50 ms. This work presents a practical and efficient solution for developing a single-camera fall detection system robust to viewpoint variations, and introduces a reusable mapping function to convert Kinect data to the MMPose format, ensuring consistent comparison with state-of-the-art models.

## 1. Introduction

The global population is undergoing a significant demographic shift, with the number of individuals aged 65 and over projected to more than double by 2050, increasing from 9.3% in 2020 to approximately 16% of the world’s population, according to the United Nations (UN) [1]. This trend is particularly pronounced in countries like Japan, where 29% of the population is expected to be over 65, underscoring the growing public health challenge of falls among the elderly. Falls are a critical concern, as the World Health Organization (WHO) [2] identifies them as the second leading cause of unintentional injury deaths globally, especially among adults over 60. With an estimated 684,000 annual fatalities, a large proportion of which occur in low- and middle-income countries, falls pose a substantial threat to public health. These events, defined as unplanned descents to the ground, can lead to severe injuries, loss of independence, and even death, especially if assistance is delayed. The high incidence of falls also places a significant economic burden on healthcare systems, with direct medical costs in the United States alone estimated at $50 billion annually. This demonstrates the critical need for effective fall detection systems to ensure timely intervention and improve outcomes for the elderly.

However, the reliability of these systems is often undermined by the challenges of real-world environments [3]. To be truly effective, a fall detection system must function accurately despite the variability of camera positions and the presence of occlusions, where a subject is partially obscured by furniture or other objects. This requires sophisticated systems that can robustly interpret visual data in diverse and cluttered settings.

This challenge is illustrated by the difficulty a standard model has in correctly classifying a fall when the camera is positioned at an unusual height or angle [4,5,6]. A model trained on a controlled, side-view dataset may fail to recognize a fall when it is viewed from a head-on or elevated perspective. Overcoming this requires either training on a massive, diverse dataset or developing models that can effectively generalize from limited data.

Our work addresses this generalization problem by focusing on improving the robustness of fall detection models to variations in camera viewpoint and occlusions. We present a novel methodology that operates on existing skeletal data, making it applicable to a wide range of pose estimation architectures used for fall detection.

Our key contributions are summarized as follows:

- We introduce a “Synthetic Viewpoint Generation” data augmentation pipeline that efficiently generates realistic occluded and rotated skeletal sequences specifically for training fall detection models, significantly enhancing robustness to camera perspective variation and stability.
- We propose a simple but effective inference-time aggregation method that leverages multiple rotated views of a single input to achieve a consensus-based final fall prediction, further enhancing model performance.
- We propose a per-joint affine transformation to convert the Kinect skeleton format to the MMPose format, enabling the model to be trained on standard Kinect datasets.
- We conduct a comprehensive ablation study to systematically quantify the impact of each of our contributions on the accuracy of fall detection.

The remainder of this paper is organized as follows: Section 2 summarizes existing research and identifies related works. Section 3 details the dataset preparation process and the proposed fall detection system. Section 4 presents our evaluation, including a comparison with a state-of-the-art approach. The discussion and key findings are presented in Section 5, and the paper concludes with a summary and directions for future research in Section 6.

## 2. Related Work

Human fall detection systems can be categorized into two main types: those based on wearable sensors and those that employ machine vision-based techniques. This section reviews both paradigms, with a specific focus on state-of-the-art machine learning and deep learning methods used for fall detection.

Wearable sensor systems typically use devices such as gyroscopes, accelerometers, pressure sensors, tilt switches, and magnetometers to capture various signals. From these signals, parameters like angles, distances, and statistical measures are directly extracted. Wearable systems for fall detection are inherently data-set-dependent for training and can be deployable across various environments. In an early study [7], researchers developed an accelerometer-based system. It first checks if the signal vector magnitude (SVMA) exceeds a given threshold. If it does, a pulse sensor and trunk angle are used for verification. This system, which automatically contacts emergency services upon fall confirmation, achieved a high accuracy of 97.5%. Another approach was seen in study [8], where a hip-worn system that used a variety of sensors with a logistic regression classifier achieved 100% accuracy in detecting falls. However, this study did not detail the variability of fall actions. A different approach [9] used a highly optimized machine learning framework with acceleration and angular velocity data for fall detection, achieving 100% accuracy using the QSVM and EBT algorithms.

Accelerometers have proven to be highly efficient in fall detection and were widely used in most systems developed. However, they also have significant disadvantages. A major issue is user compliance, as individuals may find the devices uncomfortable or forget to wear them, which compromises their effectiveness. The sensors themselves also have technical limitations, most notably a tendency for false alarms, where normal activities are mistaken for falls. Furthermore, their drawbacks include limited battery life, which can be particularly problematic for elderly people. Other significant issues involve the high cost of some devices and the associated privacy concerns about the sensitive data they collect.

In contrast, vision-based systems have been applied in various fields, including human fall detection. Vision-based systems offer a non-intrusive alternative that does not require users to wear physical devices. These systems analyze RGB and depth images from real-time video streams. This visual data is then processed using advanced deep learning algorithms to enable accurate, real-time monitoring and event classification.

The core input for many of these systems is the human skeleton, represented by a set of 2D or 3D keypoints. 3D coordinates can be obtained from low-cost depth sensors like Microsoft Kinect (Manufactured by Microsoft Corporation, Headquarters: Redmond, Washington, U.S.), or extracted from 2D images using sophisticated pose estimation algorithms powered by Convolutional Neural Networks (CNNs), such as Openpose [10] (Developed by researchers at Carnegie Mellon University, Pittsburgh, Pennsylvania, U.S.) and MMPose [11], an open-source toolbox developed by the OpenMMLab project (Headquarters: China). Early approaches for fall detection used machine learning algorithms with skeleton joint data. For example, this study [12] introduced a feature-based method that first divides the human skeleton into five parts before applying a Support Vector Machine (SVM), achieving 93.56% accuracy on the TST fall detection dataset v2. In another study, a depth camera-based system [13] analyzed the 3D trajectory of a person’s head with an SVM classifier to detect falls while checking for post-fall recovery to prevent false alarms, reporting no false positives in tests. Another work [14] used a four-camera setup, applying a median filter for background removal and morphological techniques to isolate the area of an individual before using an SVM classifier with features like head velocity, center of gravity speed and the proportion of the individual to the picture’s size. This method reported a sensitivity of 77.8% and a PPV of 36.66%. Deep learning methods, such as Convolutional Neural Networks (CNNs) and Recurrent Neural Networks (RNNs), have also been widely used. It has been shown that using multiple cameras improves performance over a single kinematic camera.

One notable approach [15] demonstrated this by combining a 3D CNN with an LSTM-based mechanism to identify crucial motion features, achieving 100% accuracy on a sports dataset and a fall detection benchmark. Similarly, another system utilized RF signals and a state machine-governed CNN, demonstrating strong performance with a recall of 94% and a precision of 92% in tests involving over 140 people [16]. A third study proposed a system that used Mask R-CNN to extract moving objects and an attention-guided Bi-directional LSTM for classification, achieving 96.70% accuracy on the UR-Fall detection dataset [17]. Furthermore, a pose-based system utilized OpenPose for precise pose estimation and a 1D-CNN to classify motion-based features extracted with a short-time Fourier transform (STFT), achieving high accuracies across multiple datasets: 99% on the UR Fall Dataset, 91% on the MCFD dataset, and 98% on the NTU RGB+D Dataset [18]. Finally, a separate system was developed combining traditional algorithms with a 1DCNN model, demonstrating high accuracy and robustness on the NTU RGB+D dataset [19]. Despite their success, initial deep learning methods for processing skeleton data, such as RNNs and CNNs, have limitations. They require pre-processing steps to re-organize 3D joint coordinates into 1D sequences or 2D pseudo-images. RNNs struggle to model the non-sequential, structural relationships between non-adjacent joints, while CNNs are constrained by their fixed-grid nature and fail to capture the intrinsic, non-Euclidean topology of the human skeleton.These limitations have driven attention toward methods based on Graph Convolutional Networks (GCNs), which are naturally suited for graph-structured data. Recent research has increasingly adopted GCNs for action recognition because these models treat the human body as a natural graph structure. This representation allows GCNs, and their spatio-temporal variants (ST-GCNs), to effectively model and capture the complex spatio-temporal dependencies between all joints simultaneously. This transition has led to state-of-the-art results in applied fields. For instance, an ST-GCN model model in [20] has been successfully employed for fall detection using skeletal data from a Kinect v2 sensor and transfer learning achieved 100% accuracy on the TST Fall v2 dataset and 97.33% on the Fallfree dataset. In related work, a GCN-based model developed by [21] achieved approximately 99% accuracy on the ImViA RU-Fall detection dataset. In another study [22], a novel three-stream system with an ST-GCN model demonstrated superior performance, achieving accuracies of 99.68%, 99.97%, 99.47%, and 98.97% on the ImViA, Fall-UP, FU-Kinect, and UR-Fall datasets, respectively. Deep learning methods are powerful for classification tasks due to their feature extraction capabilities, but they are highly contingent on the availability of large, annotated datasets. They also often exhibit vulnerability to environmental factors like occlusion and are not robust to variations in camera viewpoint. Although multi-camera setups [6,23] have been used to enhance viewpoint robustness, they require complex fusion layers, leading to significant computational demands and complicated deployment logistics [24].

To address these challenges, our work proposes the VIRA-GCN model, an adaptation of the Richly Activated Graph Convolutional Network (RA-GCNv2) for fall detection using skeleton data derived from the MMpose framework. Our approach employs a lightweight, inference-time aggregation method that focuses on robustness to real-world occlusions. Crucially, this system operates on a single input stream by generating synthetic views and aggregating their predictions, thereby eliminating the need for multiple physical cameras. This mechanism results in a fall detection system that is not only highly accurate and efficient but also robust and generalizable.

## 3. Methodology

This section outlines the entire methodology developed for robust human fall detection. The approach is organized around four critical stages: dataset preparation, feature extraction, model definition, and data transformation. The process commences with the dataset preparation stage, which defines the selected data for the experimentation and evaluation of the proposed method and includes the necessary augmentation strategies. Feature extraction then details the vital process of feature extraction and justifies the selection of the MMPose framework for obtaining robust 3D skeletal data. This is followed by a detailed description of the proposed VIRA-GCN Model Architecture. Finally, the section concludes with an explanation of the specialized Kinect to MMPose Data Transformation pipeline.

### 3.1. Dataset Preparation

A set of publicly available datasets containing both fall and non-fall actions was first identified for this study. The primary selection criterion was the availability of RGB video to enable the extraction of 3D skeletal data using the MMPose framework [11].

As summarized in Table 1, several datasets meet these requirements. The Le2i Fall Dataset was the first selected and utilized for model training and validation. This selection was motivated by its provision of the essential RGB video sequences required for 3D skeletal data extraction and its representation of complex and realistic fall scenarios, which are crucial for validating the model’s robustness to real-world conditions. The NTU RGB+D dataset was then specifically chosen for a technical validation purpose: facilitating a fair comparison between data from Kinect sensors and data processed by MMPose. Since this dataset provides both types of data, it allows for the validation of the transformation pipeline.

#### 3.1.1. Le2i Dataset

The Le2i dataset [25] contains video sequences of various fall events and normal daily activities. The dataset was recorded using a single, fixed camera setup across various indoor environments, including a home, coffee shop, lecture hall, and office. The “fall” videos typically include a sequence of consecutive actions: standing, the actual fall, and remaining fallen, often preceded by sitting, while the “normal” category features common actions such as walking, standing, squatting, and sitting. To ensure diversity, actors wore different clothing and simulated a range of activities; however, only one person is displayed in the video. All videos have a resolution of 320 × 240 pixels, a frame rate of 25 frames/s, and feature a fixed, simple background contrasted with complex image textures.

**Figure 1:**
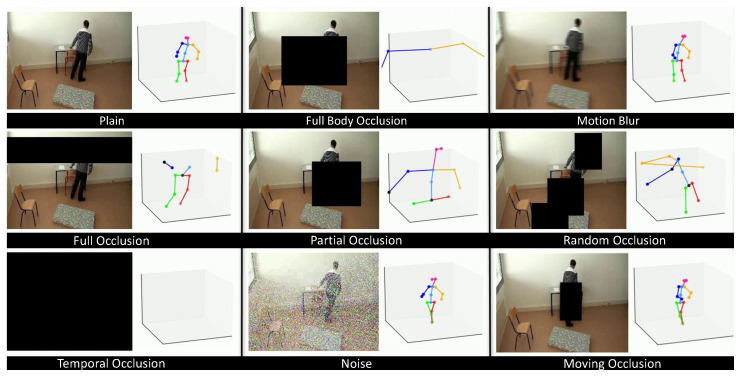
A raw image from the Le2i dataset showing a subject in an office environment. The image illustrates various types of simulated occlusions applied to the dataset to improve model robustness.

To ensure a consistent and fair evaluation, we meticulously addressed the issue of potential data leakage by first splitting the original Le2i videos into distinct training and testing subsets. The various datasets for our experiments were then created by processing these pre-defined subsets:

1. Baseline Dataset:This dataset was established by extracting the initial 3D joint coordinates from the RGB videos in both the training and testing subsets, utilizing the MMPose framework, as detailed in Section 3.2. This corpus served as the essential performance baseline for subsequent comparative analysis.
2. Occluded Dataset: This dataset was derived from the baseline by applying various types of random occlusions as illustrated in Figure 1. This method simulates realistic scenarios where a subject might be partially obscured before or during a fall. We utilized distinct occlusion modalities, including random black boxes over sequential frames and temporal occlusions, where all frames are rendered black for a short duration. From a single source video, 10 unique occluded variations were generated. The joint predictions were subsequently re-extracted from these altered frames, thereby yielding intentionally degraded coordinate data for robustness training. The resulting corpus was subjected to a fixed, random split for training and testing evaluation.
3. Rotated Dataset: This dataset was generated to enhance the model’s robustness against camera viewpoint variations. This was achieved through Synthetic Viewpoint Generation, a technique that programmatically augments each skeletal sequence by creating multiple synthetic views via 3D rotations.
  Let a skeleton sequence be represented as:
  $$\mathrm{Skel}=\{p_{t,v}\in R^{3}|t=1,...,T;v=1,...,V\}$$
  where $p_{t,v}={\left(x_{t,v},y_{t,v},z_{t,v}\right)}^{⊤}$ denotes the 3D coordinates of joint *v* at frame *t*, with T=300 frames and V=17 joints.
  We applied two-axis rotations to each sequence: a vertical (*z*-axis) rotation and a horizontal (*x*-axis) tilt. The rotation angles were defined as:
  $$\Theta_{z}=\left\{0{}^{\circ},40{}^{\circ},80{}^{\circ},120{}^{\circ},160{}^{\circ},200{}^{\circ},240{}^{\circ},280{}^{\circ},320{}^{\circ}\right\},\;\Theta_{x}=\left\{0{}^{\circ},15{}^{\circ},25{}^{\circ}\right\}.$$
  For each pair of angles $\left(\theta_{x},\theta_{z}\right)\in \Theta_{x}\times \Theta_{z}$, we constructed a combined rotation matrix:
  $$R\left(\theta_{x},\theta_{z}\right)=R_{x}\left(\theta_{x}\right)R_{z}\left(\theta_{z}\right)$$
  where
  $$R_{x}\left(\theta_{x}\right)=\left[\begin{array}{ccc} 1 & 0 & 0\\ 0 & \cos\theta_{x} & -\sin\theta_{x}\\ 0 & \sin\theta_{x} & \cos\theta_{x} \end{array}\right],\;R_{z}\left(\theta_{z}\right)=\left[\begin{array}{ccc} \cos\theta_{z} & -\sin\theta_{z} & 0\\ \sin\theta_{z} & \cos\theta_{z} & 0\\ 0 & 0 & 1 \end{array}\right]$$
  Each joint position was then transformed as:
  $$p_{t,v}^{\prime }=R\left(\theta_{x},\theta_{z}\right)p_{t,v}.$$
  This procedure generated:
  $$N_{views}=|\Theta_{x}\left|\times \right|\Theta_{z}|=3\times 9=27$$
  synthetic viewpoints per original skeleton sequence, thereby enriching the dataset and enhancing the model’s ability to generalize across different camera perspectives.
  A significant challenge we faced was the severe class imbalance within the dataset, as the number of “fall” instances was disproportionately large compared to actual “no fall” events. To address this, we applied a data augmentation strategy to the minority class. By generating additional data for “no fall” events, we created a more balanced training set, which was critical for building a robust model that performs accurately on both fall and non-fall scenarios.

#### 3.1.2. NTU RGB+D Dataset

To enable a direct and equitable comparison of our model with state-of-the-art results from literature that utilize Kinect data, we developed a per-joint affine transformation to convert the Kinect skeleton structure into the MMPose format. We utilized the NTU RGB+D dataset. This dataset is a widely recognized benchmark for human activity recognition, establishing an ideal platform to demonstrate our model’s effectiveness beyond the domain-specific constraints of Le2i.

For training and evaluation, we selected the NTU RGB+D dataset’s ’falling down’ class (labeled ’A043’) and a set of ’no fall’ actions. The ’no fall’ samples were systematically chosen from all daily action classes (A001–A042, A044–A102), including examples such as A001 (drink water), A002 (eat meal), A008 (sit down), and A024 (kicking something). The total number of selected samples was 1028, and the classes were balanced to ensure fair training.

### 3.2. Skeletal Feature Extraction

Effective feature extraction forms the foundational step of any vision-based action recognition system. For robust fall detection, this process requires the reliable acquisition of 3D skeletal data, as the third dimension (depth, z) is essential for creating viewpoint-invariant metrics such as true body height and vertical drop velocity. This capability is critical for training a model to generalize effectively against the effects of camera angle variation and partial occlusion.

Accurate implementation of this step necessitates the selection of an optimal Human Pose Estimation framework. We utilized MMPose, an open-source toolbox developed by the OpenMMLab project [11], to obtain the raw skeletal data. The justification for this selection, as summarized in the comprehensive analysis presented in Table 2, highlights MMPose’s superior alignment with our research requirements. MMPose was chosen because it facilitates the extraction of robust and accurate 3D skeletal representations from the RGB videos. This rich representation mitigates the viewpoint-dependent limitations inherent in 2D data, which is non-negotiable for training the model on synthetically augmented (occluded and rotated) inputs.

Strategically, MMPose provides the optimal balance of performance metrics. While competing frameworks like OpenPose incur a significantly high computational cost (160 GFLOPs), MMPose remains highly efficient, making it suitable for deployment with the lightweight VIRA-GCN architecture.

### 3.3. Proposed Model Architecture

This study introduces a Viewpoint Invariant Robust Aggregation Graph Convolutional Network (VIRA-GCN). This model is engineered to address the critical gap of real-world robustness by adapting the Richly Activated Graph Convolutional Network (RA-GCNv2) [36]. Our primary objective is to develop a single-camera solution that is robust to perspective variations and partial occlusions.

The VIRA-GCN architecture retains the foundational analytic strengths of its predecessor. Specifically, it leverages the Graph Convolutional Network (GCN) framework to model the human body as a graph for extracting complex spatio-temporal relationships between joints, and it utilizes the recurrent attention mechanism to dynamically weight and emphasize the most temporally relevant frames indicative of a fall event. These core capabilities remain integral to the proposed methodology.

**Figure 2:**
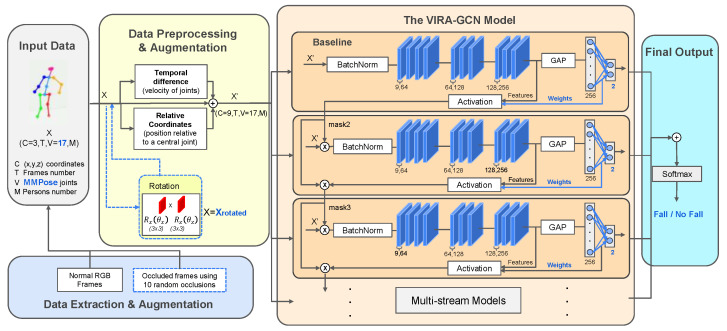
This diagram illustrates our proposed VIRA-GCN architecture for Fall Detection. We enhance its robustness to real-world conditions by introducing a novel preprocessing pipeline that augments the 17-joint input data with realistic occlusion and rotations (adapted from the original RA-GCNv2 work by [36]).

Our adapted pipeline, illustrated in Figure 2, incorporates three principal modifications to the original RA-GCNv2 architecture to enhance its practical utility and focus:

- Input Dimensionality Reduction: We adapted the input layer to process a more concise 17-joint skeleton, which aligns with modern pose estimation frameworks (e.g., MMPose). The original model was designed for the larger 25-joint skeleton from Kinect.
- Realistic Data Augmentation for Robustness: The original study employed an artificial occlusion methodology that is fundamentally limited in its capacity to represent real-world operating scenarios. This method directly altered the predicted joint values, which may not reflect real outputs from a skeletal prediction model for an occluded frame. To overcome this constraint and enhance model robustness, we implemented a comprehensive preprocessing pipeline incorporating two forms of realistic data augmentation. This strategy includes Realistic Occlusion, which simulates the partial visibility of body parts due to environmental factors, and Viewpoint Rotation, which simulates the diversity of camera perspectives encountered in naturalistic settings. As detailed in the preceding section, this deliberate augmentation process ensures the resulting model can accurately identify falls across a representative range of visual conditions, maintaining performance even when full body visibility is compromised.
- Task Re-purposing for Binary Classification: We adapted the base RA-GCNv2 architecture, transforming its original function of general action recognition into a specific binary classification task. This involved re-purposing the final layers and loss functions to distinctly discriminate between fall and non-fall events. This crucial architectural modification compels the model to concentrate its learning on the subtle, critical kinematic indicators of a fall, such as rapid changes in vertical displacement or sudden, precipitous collapses, thereby optimizing its sensitivity for the target safety application.

#### 3.3.1. Training Pipeline Optimization

We identified a major efficiency bottleneck within the initial training framework, evidenced by GPU utilization persistently restricted to approximately 30%. This computational inefficiency was attributable to the sequential execution of the data augmentation operations, specifically skeleton rotation, which occurred synchronously within the main training loop via micro-batches. Our optimization strategy resolved this limitation by refactoring the augmentation logic to be executed entirely within the dataloader. This architectural modification enabled the rotation process to be performed in parallel and decoupled from the primary training thread, ensuring the model received full batches of pre-transformed samples. This procedural change yielded a significant enhancement in throughput, substantially increasing GPU utilization, allowing for the stable use of a larger batch size of 64, and consequently accelerating the training process while maintaining a modest memory footprint of only 8 Gb. Furthermore, we integrated ‘torch.autocast’ to leverage mixed 16/32-bit precision, providing additional optimization of memory consumption and calculation speed.

#### 3.3.2. Inference-Time Aggregation

To enhance the robustness of the trained model without incurring a costly retraining process, we developed a consensus-based Inference-time Aggregation method. This approach leverages the model’s pre-trained viewpoint invariance to derive a more reliable final classification from a single input stream.

The implementation follows a defined sequential process. Firstly, multiple synthetic viewpoints of the input skeleton sequence are generated by applying a set of fixed rotations (e.g., 15°, 25°). Subsequently, each of these rotated sequences is independently processed by the trained VIRA-GCN model. The model’s prior training with rotated skeletons from multiple angles ensures it is able to handle these transformed inputs effectively. Following prediction, the model outputs an individual probability for a fall for each sequence. Finally, the probabilities derived from all rotated sequences are combined using a simple probability averaging schema to produce the final, statistically more robust fall classification.

A transformation from kinect to MMPose was proposed to ensure that the input skeleton to our model is consistent in term of joint topology and spatial alignmenet, regardless of the original data source. The core of our methodology is a per-joint affine transformation, which aligns the Kinect skeleton to the MMPose structure.

Unlike a single transformation for the entire body, our approach calculates a unique transformation matrix and translation vector for each joint. This is a more general approach that accounts for rotation, scaling, and shearing, as well as translation and is able to correct for the different joints schema.

### 3.4. Kinect to MMPose Data Transformation

The transformation for a single joint *j*, in 3D Euclidiean space, can be formally expressed as:

$$P_{transformed,j}=P_{kinect,j}\cdot A_{j}^{⊤}+b_{j}$$

**Figure 3:**
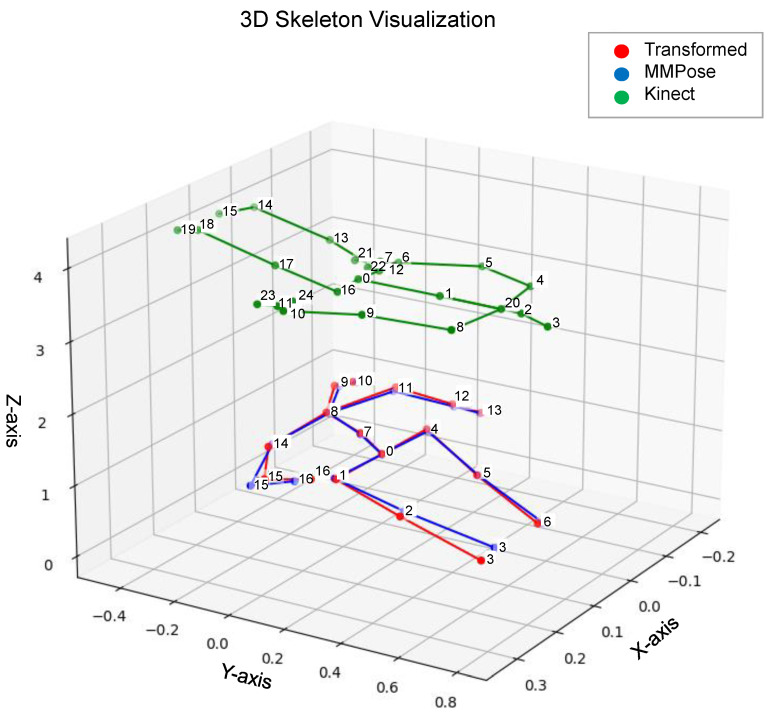
3D skeleton comparison showing the alignment achieved by the per-joint affine transformation. The original Kinect skeleton (green) is transformed to align with the target MMPose skeleton (blue), resulting in the transformed skeleton (red). The numbers displayed next to the joints represent the specific index (ID) of the joint within the respective Kinect (0-24) or MMPose (0-16) skeleton format. The mapping between these indices is detailed in the X-axis labels of Figure 4.

Here, $P_{transformed,j}$ is the 3D coordinate vector of the transformed Kinect joint, and $P_{kinect,j}$ represents the original Kinect joint’s position. Where $A_{j}$ is the unique 3×3 transformation matrix for joint *j* and $b_{j}$ is the unique 3×1 translation vector for joint *j*. The alignment achieved by this per-joint affine transformation is visually represented in Figure 3.

**Figure 4:**
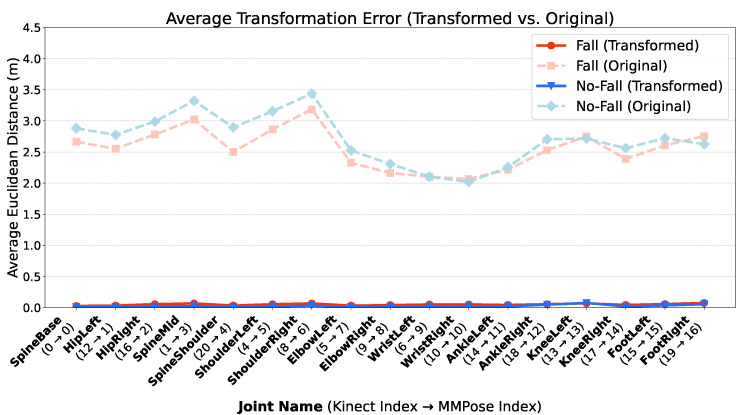
Average Euclidean Distance Error per Joint. The bar chart compares the mean Euclidean distance error for each of the 17 joints between the ground-truth MMPose skeleton and the original Kinect skeleton versus the transformed Kinect skeleton.

The transformation parameters ($A_{j}$ and $b_{j}$) are derived from a training dataset of 30 randomly selected frames. These parameters are calculated for each joint by solving a least-squares problem, which finds the optimal transformation that minimizes the distance between the training points of the Kinect and MMPose data. The performance of our model is quantitatively evaluated using the Mean Squared Error (MSE). The MSE for a single joint *j* is calculated as the average squared Euclidean distance between the transformed and target joints across the training set.

The Euclidean distance, along with its squared form, the Mean Squared Error (MSE), plays a central role in the transformation methodology as it provides both a physically meaningful error measure and the mathematical properties necessary for optimization. For 3D pose data, the Euclidean distance is the most direct and geometrically intuitive metric, quantifying the actual shortest physical displacement between the transformed joint and the ground-truth target joint. Crucially, the squared distance results in a smooth and differentiable MSE loss function, which is essential for the effective application of least-squares optimization to analytically determine the optimal transformation parameters ($A_{j}$ and $b_{j}$). Moreover, the MSE metric is the established standard metric in the field, often referred to as the Mean Per Joint Position Error (MPJPE), ensuring that the quantitative evaluation of the model’s positional accuracy is comparable with existing research.

$$MSE_{j}=\frac{1}{N_{train}}\sum_{i=1}^{N_{train}}{∥P_{mmpose,j,i}-P_{transformed,j,i}∥}_{2}^{2}$$

The overall model performance is then measured by the average MSE across all joints:

$$\mathrm{Overall}\;\mathrm{MSE}=\frac{1}{N_{joints}}\sum_{j=1}^{N_{joints}}MSE_{j}$$

## 4. Experiments and Results

This section systematically presents the empirical evaluation and quantitative results of the proposed fall detection framework. Our evaluation was rigorously structured as a comprehensive ablation study, designed to systematically quantify the isolated impact of data augmentation and, critically, to compare the generalization capabilities of our models when trained on single-domain versus combined-domain data.

### 4.1. Experimental Design

The rigorous assessment of the VIRA-GCN model’s generalization capabilities and robustness necessitated a comprehensive experimental design, centred on controlling for both data composition and stochastic variability in model initialization. To ensure the statistical reliability of the performance metrics, all model configurations underwent a 5-fold cross-validation procedure, which was independently repeated across 5 distinct random seeds. This methodology yielded an aggregate of 25 independent training and evaluation trials for each configuration, allowing for a robust estimation of mean performance and variance.

#### 4.1.1. Evaluation Datasets

The performance and cross-domain generalization capabilities of the models were rigorously assessed against a comprehensive suite of five distinct evaluation datasets, each serving a specific analytical purpose. All evaluations were conducted on the dedicated, non-overlapping held-out test subsets of the respective data sources. This methodical approach allowed for the isolation of factors such as data augmentation efficacy, robustness to corruption, and domain shift resilience. The datasets employed are detailed as follows:

- Plain (Le2i): This dataset comprises the raw, unaugmented 3D skeleton joint sequences extracted directly from the Le2i video corpus using the MMPose. Evaluation on this set established the baseline performance of the models on the native, clean data of the source domain.
- Occluded (Le2i): Consisting of skeleton joints derived from Le2i videos that were intentionally subjected to realistic visual occlusions, this set utilizes the MMPose format. Performance here directly quantifies the model’s inherent robustness against common real-world data corruptions, specifically in the form of partial data visibility.
- Rotated (Le2i): This set is composed of 3D skeleton sequences from the Le2i test set that were synthetically transformed via viewpoint rotations. Adhering to the MMPose format, this evaluation measures the model’s invariance to synthetic changes in camera perspective within the source domain.
- Mntuplain (NTU MMPose): This dataset features 3D skeleton joints extracted from the original NTU RGB+D videos using the MMPose framework. Evaluation on this set provides a direct measure of the model’s generalization performance when confronted with the standard skeleton format of the target domain.
- Kntuplain (NTU Kinect Transformed): This is a critical cross-domain evaluation set comprising 3D skeleton joints that were transformed from the original NTU RGB+D Kinect sensor data into the MMPose Transformed format using the proposed affine transformation pipeline. Performance on this dataset is essential for assessing the efficacy of the proposed transformation methodology in aligning the characteristics of the NTU Kinect data with the model’s training space, providing insight into robustness against sensor heterogeneity.

#### 4.1.2. Training Model Variants

The training phase was structured around four distinct model configurations, carefully named to reflect the specific scope of their training data and designed to isolate the impact of progressive data augmentation and domain expansion:

- ${\mathrm{VIRA-GCN}}_{\mathrm{Base}}$ (Model Le2i-Plain): This preliminary model was trained exclusively on the Plain subset of the Le2i skeleton data. It serves as a fundamental reference point, establishing the performance achievable without the introduction of any data augmentation.
- ${\mathrm{VIRA-GCN}}_{\mathrm{Rob}}$ (Model Le2i-Occluded): This second preliminary model expanded the training set to include both the Plain and the Occluded sequences from the Le2i dataset. Its purpose was to quantify the incremental robustness gained solely from simulating occlusions.
- ${\mathrm{VIRA-GCN}}_{\mathrm{Spec}}$ (Model Le2i-FullAug): This primary model was trained exclusively on a highly augmented version of the Le2i dataset, explicitly combining the raw skeleton data Plain, sequences subjected to realistic visual occlusions Occluded, and sequences augmented with synthetic viewpoint rotations Rotated. This configuration used the Le2i data augmented across all defined transformation categories. The primary purpose of this model was to establish the maximum performance and robustness achievable solely within the well-controlled Le2i domain and to serve as a critical baseline to quantify the generalization failure, or domain shift, when evaluated against the distinct NTU RGB+D dataset.
- ${\mathrm{VIRA-GCN}}_{\mathrm{Gen}}$ (Model Le2i-NTU-DomainAug): This model was introduced to specifically address the identified challenge of cross-dataset generalization. It significantly expands the training regime by incorporating augmented data from both the Le2i and the NTU RGB+D domains. It retained the comprehensive Le2i augmented training set and further included the Kinect-transformed data Kntuplain and its synthetic viewpoint rotations Rotated Kntuplain derived from the NTU dataset. By explicitly exposing the model to the characteristics of both datasets, this model was engineered to achieve robust generalization across heterogeneous data sources, aiming to bridge the performance gap observed in cross-domain evaluation.

### 4.2. Transformation Results

We evaluated the effectiveness of our per-joint affine transformation from the Kinect to the MMPose skeleton format. Figure 3 shows a visual comparison of the transformed skeleton, demonstrating an impressive alignment with the target MMPose structure. Quantitatively, the Mean Squared Error (MSE) chart, as confirmed by the visualization in Figure 4, demonstrates that our transformation method significantly reduces the Euclidean distance error for each joint, bringing the Kinect data much closer to the MMPose format.

### 4.3. Comparative Model Performance

The models were evaluated using four standard performance metrics: accuracy, sensitivity, specificity, and F1-score, all calculated from the confusion matrix elements: true positives (TP), true negatives (TN), false positives (FP), and false negatives (FN). Fall sequences were consistently labeled as positive samples, and normal daily activities as negative samples:

(1) $$\mathrm{Accuracy}=\frac{TP+TN}{TP+TN+FP+FN}$$

(2) $$\mathrm{Precision}=\frac{TP}{TP+FP}$$

(3) $$\mathrm{Sensitivity}=\frac{TP}{TP+FN}$$

(4) $$\mathrm{Specificity}=\frac{TN}{TN+FP}$$

(5) $$F1-\mathrm{score}=2\cdot \frac{\mathrm{Precision}\cdot \mathrm{Sensitivity}}{\mathrm{Precision}+\mathrm{Sensitivity}}$$

**Figure 5:**
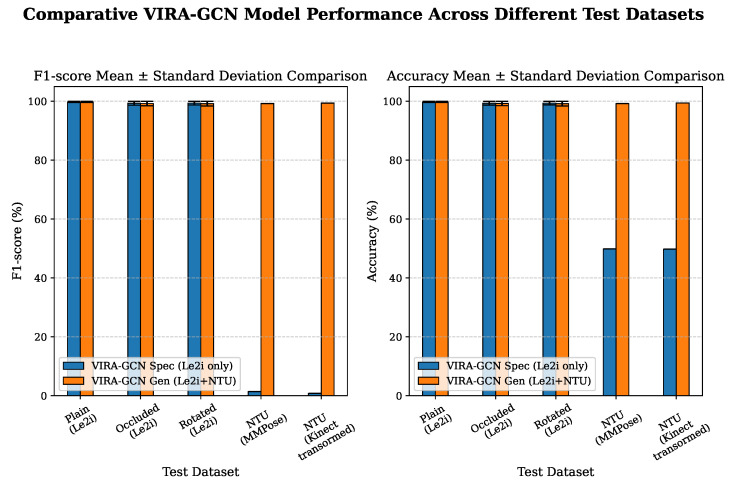
Comparative Model Performance Across Different Test Datasets. The figure shows the F1-score and accuracy (Mean ± Standard Deviation across 25 runs) comparing ${\mathrm{VIRA-GCN}}_{\mathrm{Spec}}$ and ${\mathrm{VIRA-GCN}}_{\mathrm{Gen}}$ when evaluated on five distinct test subsets: Plain, Occluded, Rotated (Le2i domain), and Mntuplain, Kntuplain (NTU domain).

The decisive comparison between the models incorporating different degrees of domain information is visually synthesized in Figure 5, which plots the F1-score and accuracy across all five test datasets. The results demonstrate the critical impact of integrating target domain data into the training regimen.

#### 4.3.1. Initial Augmentation Impact (Le2i-Plain Dataset)

To isolate the contribution of augmentation techniques, the initial model variants were evaluated. Table 3 details the comparative performance of the models when tested exclusively on the unaugmented (Plain) Le2i dataset.

The data demonstrates a critical dependency on augmentation strategy, even for canonical views. The strictly ${\mathrm{VIRA-GCN}}_{\mathrm{Base}}$ exhibited a measurable deficiency, achieving a 94.52% F1-score, 94.25% accuracy, and a low mean sensitivity of 90.34%. In contrast, both augmentation strategies led to substantial performance gains. ${\mathrm{VIRA-GCN}}_{\mathrm{Rob}}$ achieved a 99.50% F1-score, 99.69% accuracy, and 99.25% sensitivity, while ${\mathrm{VIRA-GCN}}_{\mathrm{Spec}}$ attained the highest metrics overall, with a 99.81% F1-score and 99.81% accuracy. Furthermore, the perfect mean specificity (100%) achieved by both augmented models ${\mathrm{VIRA-GCN}}_{\mathrm{Rob}}$ and ${\mathrm{VIRA-GCN}}_{\mathrm{Spec}}$, confirms that the feature learning derived from augmentation successfully eliminates false positives in this controlled test scenario, underscoring its general benefit to feature robustness.

#### 4.3.2. Performance on Le2i Domain Datasets

On the Le2i domain test sets (Plain, Occluded, Rotated), both primary models: the ${\mathrm{VIRA-GCN}}_{\mathrm{Spec}}$ and the ${\mathrm{VIRA-GCN}}_{\mathrm{Gen}}$ achieved exceptionally high performance, with metrics consistently approximating 100%. This sustained, near-perfect performance confirms the efficacy of the initial Le2i data augmentation strategy for achieving feature robustness within the source domain.

Specifically, ${\mathrm{VIRA-GCN}}_{\mathrm{Spec}}$ confirmed the effectiveness of the initial Le2i-only augmentation strategy for viewpoint robustness, achieving 99.38% accuracy and 99.37% F1-score on the Rotated test set. Crucially, the ${\mathrm{VIRA-GCN}}_{\mathrm{Gen}}$ maintained this high performance across all Le2i subsets, demonstrating that the inclusion of auxiliary NTU training data did not negatively impact the model’s specialized performance or feature discrimination within the original source domain.

For the subsequent comprehensive cross-domain evaluation, we focus on the two highest-performing configurations: the ${\mathrm{VIRA-GCN}}_{\mathrm{Spec}}$ (Model Le2i-FullAug) and the ${\mathrm{VIRA-GCN}}_{\mathrm{Gen}}$ (Model Le2i-NTU-DomainAug), which were evaluated against all five test subsets, as detailed in Table 4.

#### 4.3.3. Performance on NTU Domain Datasets

The decisive divergence in model efficacy is rigorously documented on the NTU domain test sets, specifically Mntuplain and Kntuplain. The ${\mathrm{VIRA-GCN}}_{\mathrm{Spec}}$, trained exclusively on the Le2i dataset, exhibited a pronounced inability to generalize to the NTU domain. Consistent with initial hypotheses, this configuration registered F1-scores and accuracy metrics approximating 50%. This result unequivocally substantiates the existence of a severe domain shift between the proprietary Le2i data and the publicly sourced NTU dataset characteristics.

Conversely, the **${\mathrm{VIRA-GCN}}_{\mathrm{Gen}}$** successfully mitigated this domain generalization deficit. This model achieved near-optimal performance across both NTU evaluation subsets:

- Mntuplain (NTU MMPose): F1-score approximated 99.25% and accuracy approximated 99.22%.
- Kntuplain (NTU Kinect Transformed): F1-score approximated 99.40% and accuracy approximated 99.42%.

Furthermore, the perfect mean specificity (100%) attained by the ${\mathrm{VIRA-GCN}}_{\mathrm{Gen}}$ on the NTU test sets confirms that the model successfully acquired the discriminatory features necessary to differentiate between the specific fall dynamics inherent to the NTU domain and general non-fall activities, thereby demonstrating robust cross-domain generalization.

### 4.4. Computational Analysis and Hardware Configuration

To substantiate the claim that the VIRA-GCN model is suitable for low-resource deployment, we provide a rigorous quantitative analysis of both its complexity and hardware performance. The comprehensive metrics for model size, computational demand, and inference latency are detailed in Table 5.

The system exhibits high computational efficiency, utilizing 4.06 M parameters and requiring 7.85 GFLOPs. Inference speed, a critical metric for real-time suitability, was measured on an NVIDIA RTX 3090 GPU (Manufactured by NVIDIA Corporation, Headquarters: Santa Clara, California, U.S., 24GB VRAM, 32 GB RAM). The average latency of 7.50 ms per sample is notably well below the typical frame interval of video streams (∼33ms for 30FPS). These quantitative results affirm that the model is lightweight and fully capable of real-time inference, supporting its suitability for deployment in resource-constrained settings.

A key factor contributing to this high overall efficiency was the optimization of the training pipeline. Initially, the pipeline was highly inefficient, leading to GPU utilization restricted to approximately 30%. We resolved this bottleneck by refactoring the data augmentation logic to be executed entirely within the dataloader, enabling the process to run in parallel. This crucial architectural modification drastically improved GPU utilization and accelerated the training process.

## 5. Discussion

The empirical findings from the comparative study between the training configurations validate that utilizing a combined, heterogeneous, and augmented dataset is the most effective strategy for building a truly robust and generalizable fall detection model. While Model ${\mathrm{VIRA-GCN}}_{\mathrm{Spec}}$ demonstrated exceptional performance within its source domain, achieving near-perfect scores on the Plain, Occluded, and Rotated Le2i test sets, its severe performance drop to approximately 50% on the NTU domain test sets (Mntuplain, Kntuplain) unequivocally confirmed the critical problem of domain shift.

### 5.1. The Critical Challenge of Domain Shift

**Figure 6:**
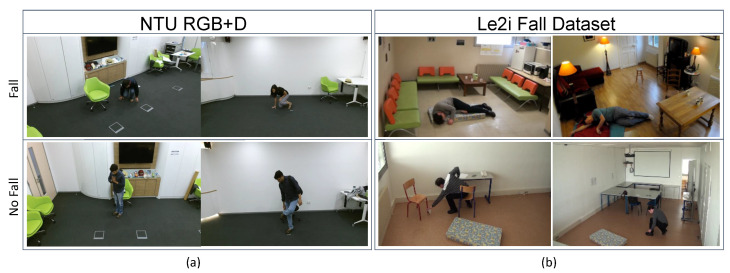
Comparative analysis of fall and non-fall actions in the (**a**) NTU RGB+D and (**b**) Le2i datasets. The Le2i dataset presents greater complexity by including non-fall actions that are visually similar to falls in NTU RGB+D, posing a significant challenge for detection models. The NTU dataset, by contrast, contains more structured and controlled fall events.
- Data Transformation Efficacy: Although our per-joint affine transformation successfully established a consistent mapping function from the Kinect to the MMPose format (Figure 4), the resultant poor cross-domain performance of ${\mathrm{VIRA-GCN}}_{\mathrm{Spec}}$ confirmed that failure was solely attributable to the intrinsic domain differences in fall dynamics, rather than mere data format misalignment. The transformation was necessary, but not sufficient, to bridge the domain gap.
- Implication for Generalization: These results suggest that standard models are prone to overfitting to specific recording conditions and sensor modalities. The successful strategy implemented in the following section demonstrates that the solution must involve explicit domain exposure.

The initial evaluation, highlighted by the performance disparity of ${\mathrm{VIRA-GCN}}_{\mathrm{Spec}}$, revealed a significant cross-dataset generalization deficit that defines a critical challenge for skeleton-based fall detection systems. This outcome confirms that performance degradation is attributed to a severe domain shift and inherent differences in the characteristics of the fall events. The main distinctions contributing to this deficit are summarized as follows:

- Fall Action Heterogeneity: As qualitatively illustrated in Figure 6, the NTU dataset generally features slower, more controlled descents where subjects often remain centered. Conversely, the Le2i dataset captures more realistic and complex scenarios, including falls initiated during activities such as walking, and subjects remain prone for the duration of the sequence. The ${\mathrm{VIRA-GCN}}_{\mathrm{Spec}}$ overfit to the complex dynamics of Le2i and could not reliably recognize the subtler, stylized falls of the NTU domain.

### 5.2. Overcoming the Generalization Deficit

${\mathrm{VIRA-GCN}}_{\mathrm{Gen}}$ successfully overcame this generalization deficit. By incorporating the transformed Kinect data (Kntuplain) and its rotated synthetic views into the training pipeline, the model achieved scores of nearly 100% on both the Le2i and the NTU test subsets. This outcome supports two fundamental conclusions:

- Domain Adaptation through Augmentation: The inclusion of the augmented NTU data successfully taught the model to recognize the specific fall characteristics and environmental conditions inherent in the NTU dataset (e.g., slower falls, post-fall recovery actions). This demonstrates that model performance degradation is not an inherent flaw in the model architecture (VIRA-GCN), but rather a failure to generalize due to domain-specific feature scarcity in the original training data.
- Efficacy of the Transformation Pipeline: The fact that ${\mathrm{VIRA-GCN}}_{\mathrm{Gen}}$ performed equally well on both the MMPose-extracted NTU data (Mntuplain) and the Kinect-transformed NTU data (Kntuplain) provides strong validation of our per-joint affine transformation method. It confirms that the transformation creates a consistently aligned skeleton format, enabling seamless aggregation of different data sources (Kinect vs. MMPose) for robust cross-domain training.

### 5.3. State-of-the-Art Performance

As summarized in Table 6, the proposed ${\mathrm{VIRA-GCN}}_{\mathrm{Gen}}$ achieves superior performance compared to previously established state-of-the-art approaches on the Le2i dataset.

Specifically, the model demonstrates dominant accuracy, reaching 99.81%, surpassing all comparable methods. Furthermore, the perfect 100.00% specificity indicates that the model exhibits zero false positives on the evaluation data, a crucial factor for reducing unnecessary alerts in real-world application systems. This state-of-the-art result validates the architectural benefits of VIRA-GCN when coupled with our comprehensive augmentation and domain adaptation strategy.

In conclusion, the VIRA-GCN model, when trained with the comprehensive augmentation strategy of Model ${\mathrm{VIRA-GCN}}_{\mathrm{Gen}}$, not only maintains its real-time efficiency and robustness to occlusions and viewpoint changes within the primary domain (Le2i) but also successfully generalizes to the challenging, distinct domain of the NTU RGB+D dataset. This robust generalization capability is achieved through a practical and efficient pipeline that seamlessly integrates different sensor data types via our novel per-joint affine transformation.

## 6. Conclusions

In this study, we presented a comprehensive methodological framework to significantly enhance the robustness and generalization of vision-based fall detection models against two major real-world limitations: occlusions and variations in camera viewpoint. The core contribution is the development of the Viewpoint Invariant Robust Aggregation Graph Convolutional Network (VIRA-GCN), which utilizes a combination of a carefully designed data augmentation strategy, namely synthetic viewpoint generation, and an efficient inference-time aggregation method, which is consensus-based prediction, to achieve substantial performance improvements without necessitating resource-heavy multi-camera setups.

The efficacy of our approach was quantitatively confirmed on the challenging Le2i dataset, where the VIRA-GCN achieved superior performance metrics, including 99.81% accuracy, 99.62% sensitivity, and 100.00% specificity. Furthermore, the model is inherently lightweight, utilizing only 4.06M parameters and achieving an average inference latency of 7.50ms per sample on an NVIDIA RTX 3090 GPU, confirming its suitability for real-time, low-resource deployment.The model’s efficiency was further established by the successful optimization of the training pipeline, which eliminated the initial computational bottleneck of restricted GPU utilization.

Crucially, the ${\mathrm{VIRA-GCN}}_{\mathrm{Gen}}$ successfully demonstrated robust cross-domain generalization. By leveraging the Kinect to MMPose transformation pipeline and incorporating augmented NTU data, the model not only maintained its high performance on the Le2i domain but also successfully generalized to the challenging, distinct characteristics of the NTU RGB+D dataset, achieving scores of nearly 100% on all NTU evaluation subsets. This finding, while confirming that model degradation is initially driven by domain shift stemming from inherent differences in fall characteristics (e.g., fast versus slow falls), validates that the problem can be mitigated through our proposed architecture and comprehensive domain adaptation strategy. In summary, this research successfully provides a valuable and computationally practical framework for developing more reliable fall detection systems that are robust to variations in camera perspective, thereby advancing their readiness for deployment in real-world applications.

## Figures and Tables

**Table 1:** A Comparative Analysis of Video Modalities and Feature Sets in Fall Detection Datasets.

Dataset	RGB Videos	Kinect Data	Viewpoint Variation	Recording Environment
Le2i Fall Dataset [25]	✔		✔	Indoor Home
UR Fall Detection [26]	✔	✔		Indoor Lab
SDUFall [27]	✔	✔		Indoor Home
SisFall [28]				Indoor Home
NTU RGB+D [24]		✔	✔	Indoor Lab
TST Fall Detection [29]	✔	✔		Indoor Lab
KFall Dataset [30]		✔		Indoor Home
MobiAct [31]				Indoor Lab
FallAllD [32]	✔			Uncontrolled
FARSEEING [33]				Uncontrolled

**Table 2:** A Comparative Analysis of State-of-the-Art Human Pose Estimation Frameworks.

Framework	Year	Target Scope	Nb Extracted Joints	Accuracy AP (%)	Cost (GFLOPs)	Efficiency
OpenPose [10]	2017	Multi-person 2D/3D Pose	15–25 up to 135	61.8	160	Slowest (GPU-Intensive)
AlphaPose [34]	2017	High-Accuracy 2D/3D Pose	17 up to 136	73.3	5.91 → 15.99	Fast (Requires GPU)
MediaPipe [35]	2019	Real-time Mobile Pipelines	33	-	29.3 → 35.4	Extremely Fast on Mobile/CPU
MMPose [11]	2020	SOTA Toolkit Benchmark	17 up to 133	75.8	1.93	90+ FPS CPU/ 430+ FPS GPU

**Table 3:** Comparative Performance of Preliminary Model Variants Evaluated on the Plain (Unaugmented) Le2i Test Dataset.

Model Name	Test Type	F1-Score Mean (%)	Accuracy Mean (%)	Sensitivity Mean (%)	Specificity Mean (%)
${\mathrm{VIRA-GCN}}_{\mathrm{Base}}$	Plain	94.52	94.25	90.34	99.09
${\mathrm{VIRA-GCN}}_{\mathrm{Rob}}$	Plain	99.50	99.69	99.25	100.00
${\mathrm{VIRA-GCN}}_{\mathrm{Spec}}$	Plain	99.81	99.81	99.62	100.00

**Table 4:** Comprehensive Comparative Performance of Primary Model Variants Grouped by Evaluation Domain.

Model Name	Test Type	F1-Score Mean (%)	Accuracy Mean (%)	Sensitivity Mean (%)	Specificity Mean (%)
${\mathrm{VIRA-GCN}}_{\mathrm{Spec}}$	Plain	99.81	99.81	99.62	100.00
	Occluded	99.34	99.35	98.70	100.00
	Rotated	99.37	99.38	98.75	100.00
	Mntuplain	1.38	49.84	0.70	99.00
	Kntuplain	0.79	49.79	0.40	99.00
${\mathrm{VIRA-GCN}}_{\mathrm{Gen}}$	Plain	99.81	99.81	99.62	100.00
	Occluded	99.24	99.25	98.50	100.00
	Rotated	99.19	99.20	98.40	100.00
	Mntuplain	99.25	99.22	99.00	99.50
	Kntuplain	99.40	99.42	99.20	99.60

**Table 5:** VIRA-GCN Computational Complexity and Performance Metrics.

Reference Hardware	Model Parameters	Computational Complexity	Inference Latency
NVIDIA RTX 3090	4.06 M	7.85 GFLOPs	7.50 ms/sample

**Table 6:** Performance evaluation of various state-of-the-art methods on the Le2i dataset.

Model Methodology	Year	Accuracy/%	Sensitivity/%	Specificity/%
Lightweight ST-GCN [37]	2021	96.10	92.50	95.70
V2V-PoseNet [38]	2021	93.67	100.00	87.00
YOLOv7-DeepSORT [39]	2023	94.50	98.60	97.00
Alphapose ST-GCN [40]	2023	98.70	97.78	100.00
3D Multi-Stream CNNs [41]	2023	99.44	99.12	99.12
Three-Stream ST-GCN [42]	2024	99.64	97.25	-
VIRA-GCN (Ours)	2025	99.81	99.62	100.00

## Data Availability

The data that support the findings of this study are available from the original sources: Le2i Fall Detection Dataset and the NTU RGB+D Dataset. The core datasets generated and analyzed during the current study, including all model performance metrics and transformation results, are contained within this published article (Table 1, Table 2, Table 3, Table 4 and Table 5 and Figure 1, Figure 2, Figure 3, Figure 4, Figure 5 and Figure 6).
